# “Just Get on with It”: Qualitative Insights of Coming to Terms with a Deteriorating Body for Older Women with Osteoarthritis

**DOI:** 10.1371/journal.pone.0120507

**Published:** 2015-03-17

**Authors:** Melissa L. Harris, Julie E. Byles, David Sibbritt, Deborah Loxton

**Affiliations:** 1 Priority Research Centre for Gender, Health and Ageing, Faculty of Health and Medicine, University of Newcastle, Callaghan, NSW, Australia; 2 Faculty of Health, University of Technology Sydney, Broadway, NSW, Australia; Scottish Collaboration for Public Health Research and Policy (SCPHRP), UNITED KINGDOM

## Abstract

**Objective:**

To qualify the psychosocial burden of osteoarthritis for older women and identify factors perceived to assist with psychological adjustment to the disease.

**Methods:**

Women who indicated being diagnosed/treated for osteoarthritis in the previous three years in the fifth survey of the Australian Longitudinal Study on Women’s Health provided the sampling frame. Participants were randomly sampled until saturation was reached using a systematic process. Thematic content analysis was applied to the 19 semi-structured telephone interviews using a realist framework.

**Results:**

The findings indicate that the emotional burden of osteoarthritis is considerable, and the process of psychological adjustment complex. Older women with osteoarthritis have psychological difficulties associated with increasing pain and functional impairment. Psychological adjustment over time was attributed primarily to cognitive and attitudinal factors (e.g. stoicism, making downward comparisons and possessing specific notions about the cause of arthritis). This was a dynamic ‘day to day’ process involving a constant struggle between grieving physical losses and increasing dependence amidst symptom management.

**Conclusion:**

The findings of this study add to the current understanding of the complex processes involved in psychological adjustment over time. Targeted interventions focused on assisting women with arthritis redefine self-concepts outside the confines of caring responsibilities, coupled with public health education programs around understanding the destructive nature of arthritis are required. Understanding the destructive and (potentially) preventable nature of arthritis may facilitate early detection and increased uptake of appropriate treatment options for osteoarthritis that have the ability to modify disease trajectories.

## Introduction

Arthritis is one of the most prevalent and pervasive chronic conditions affecting middle-aged and older adults [1,2]. It contributes substantially to global healthcare expenditure and is a major cause of disability, limited mobility and chronic pain [3,4]. The condition disproportionately affects women and when present post-menopause is more debilitating in this population [5,6]. Osteoarthritis (OA) is the most common form of arthritis [7], representing 60–70% of joint disease diagnoses [8]. Although any joint may be affected by OA, it primarily affects weight-bearing joints such as the knee, hip and spine, with the neck, feet and hand regions also implicated [9]. Localised OA (confined to a single joint) most commonly affects the distal and proximal interphalangeal joints of the hand and is largely asymptomatic [10,11]. OA is progressive and destructive in nature, leading to significant pain and loss of joint function, particularly for women [6,12]. Approximately 80% of people with OA experience limitations in movement, with 25% unable to perform major daily duties [13]. Meanwhile, approximately 10% of people aged over 60 are disabled as a result of the disease [4,14].

The pervasive nature of pain and limitation has a significant impact on psychological functioning [15]. While the majority of research has focused on rheumatoid arthritis (RA) (largely owing to perceptions that it is a more insidious disease) [16], there is evidence to suggest that OA is also accompanied by psychological distress, particularly when focused on women [17,18]. In a study of consecutive patients attending a rheumatology clinic, Axford and colleagues [19] found that almost half of patients with lower limb OA had some form of psychological distress (either depression alone, anxiety alone or both). Likewise, Marks [20] found that around 20% of community-dwelling individuals with mild to moderate knee OA had undiagnosed clinical levels of depression. Importantly however, in a ten year study involving both RA and OA outpatients (n = 6,153), Hawley and Wolfe [21] found that psychological distress was not a specific feature of RA but representative of rheumatology patients. Despite this, understanding how individuals with arthritis psychologically adjust (defined as a healthy rebalancing to new circumstances) [22] to the disease has almost exclusively focused on RA. These findings have produced conflicting results with some suggesting a reduction in distress over time [23,24], while others have found that mental health remains relatively stable over the disease course [25]. Meanwhile, a Swedish longitudinal study of primary care patients (64% female) found that a latent effect may be associated with psychological adaptation [24].

Findings from qualitative research however suggest that adjustment to arthritis is complex and involves a spectrum of emotions ranging from frustration and annoyance to fear, anger, resentment, misery and helplessness [26–28]. Particularly, in one U.S. study of older women (n = 18) with OA (aged 65–92 years), arthritis was described as an ever-present and unwelcomed entity which dominated their attention. This feeling was likened to “wearing a heavy garment” [26]. In contrast, findings from an RA-focused qualitative study of Austrian rheumatology outpatients, suggested that over time individuals who had retired as a result of their condition described their experience as positive, with the disease seen as a challenge and facilitator of personal growth [29]. Meanwhile, others have suggested that adjusting to a chronic illness is a dynamic process, influenced not only by the disease but by the individual’s life circumstances and personal resources [30]. Information about what factors women consider important to their long-term adjustment to OA is currently lacking, despite the large body of literature concentrated on the day to day management of chronic pain and physical dysfunction [31,32]. While previous research has been largely RA-focused, the findings suggest that psychosocial factors may be pivotal in this process [23].

With factors such as living a ‘normal’ life and maintaining a sense of independence viewed as important treatment goals by individuals with arthritis [33], greater attention is required regarding understanding factors that may facilitate, or provide a barrier to adaptation in OA. Capturing the depth and breadth of emotion is difficult to achieve using quantitative approaches. Therefore, the overarching aim of this study is to qualify the psychosocial impact of OA for older women and identify factors perceived to assist with adjustment to the disease.

## Materials and Method

### Ethical statement

Ethical approval was granted by the University of Newcastle’s Human Research Ethics Committee prior to the commencement of data collection, with all interviews (including verbal consent processes) carried out in accordance with the University of Newcastle’s Human Research Ethics Committee policies regarding telephone interviewing.

### Participant sampling frame

Participants were sampled from the 1946–1951 cohort of the Australian Longitudinal Study on Women’s Health (ALSWH) [34,35], a nationally representative study funded by the Australian Government since 1996. As there are inherent difficulties associated with reporting specific arthritis forms in epidemiological research [36], women who indicated on the fifth survey (conducted in 2007), that they had been diagnosed or treated for arthritis in the previous three years were included in the initial sampling frame (n = 3088/10,638). While all women reporting a primary diagnosis of OA were eligible to participate, women who did not speak fluent English, had a proxy such as a carer complete their surveys, or had withdrawn from either the longitudinal study or further sub-studies were excluded (n = 286). Potential participants were randomly selected from the remaining 2,802 women by the ALSWH data manager. It was anticipated that up to 30 women would be required to meet the study objectives. Assuming a 50% response rate, an initial sample of 60 women was drawn from the participant pool. Women however were sampled until data saturation had been reached (i.e. no new or relevant information emerging from the interviews would further develop a conceptual theme). Key demographic and health-related factors were monitored throughout the interview process to achieve a diverse sample. These included education, area of residence, Body Mass Index, physical activity and health-related quality of life (as measured by the SF-36 [37] subscales).

### Recruitment

Potential participants randomly selected from the study participant pool were mailed a letter of invitation/participant information statement by the ALSWH. As the final sample size was determined by reaching the point of data saturation, invitations were sent out in small batches (primarily ten). Potential participants were contacted by the research team (MLH) two to four weeks following the mailout in order to gain consent and clarify primary diagnosis of OA.

### Interview process

The semi-structured telephone interviews were conducted by the first author (MLH), a PhD candidate with a background in psychology who had no personal experience of arthritis and was not within the sampled age bracket. Motivation for this research revolved around an interest in untangling the relationship between psychosocial factors and chronic health conditions. All semi-structured interviews were conducted at the Research Centre for Gender, Health and Ageing, University of Newcastle and digitally recorded. Prior to the commencement of the interview questions, with the tape recorder turned on, participants provided verbal consent for the interview by answering a series of questions (e.g. Do you understand why this research is being conducted and have you had all of your questions about the research answered?; Do you consent to participate in a telephone interview about your experiences with having arthritis?). The provision of informed verbal consent was deemed more appropriate than written consent as this would reduce the unnecessary physical burden on women with a chronic pain condition having to reply via mail. This would provide the most representative sample of women with arthritis.

The interviews were primarily guided by the interview schedule, with all anchor questions being asked. Participants however were able to direct the conversation within these areas of interest and concentrate in-depth on issues they felt were most important. During the interview period, field notes were collected. These focused on points of interest to follow-up at an appropriate time and the emotional condition of the participant, among others. Following cessation of the interview, the field notes generated during the interview process were amended and further reflections and impressions (e.g. rapport generated between the researcher and participant, the quality of the data produced) and a summary of significant findings were recorded. Interviews ranged in duration from 15 mins to 2 hours 50 mins, with an average time of 1 hour 10 mins.

### Semi-structured interview schedule

The semi-structured interviews were retrospective in nature. The interview schedule (see S1 Table) was designed to guide participants towards reflecting upon their experiences with OA. Consistent with a realist-orientated approach (i.e. aiming to explain the phenomenon with a degree of objectivity) [38], the main interview questions were anchored to the research questions (e.g. “How has your life been affected by being diagnosed with arthritis?”) were open-ended in order to allow the participant to tell *their* story without direction from the interviewer. A series of prompts related to the research aims such as “How has having arthritis affected you physically? Emotionally? With your relationships with friends and family?” were also incorporated to aid discussion. While the main questions and associated prompts provided a certain amount of structure in order to conduct the interview, they were not prescriptive. To provide the contextual material necessary to understand the phenomenon, the content of the interview was ultimately co-determined by both the researcher and participant.

### Data analysis

Women were sampled until ‘data saturation’ had been reached using a systematic process similar to Francis and colleagues [39]. Briefly, a sample size of 15 was set with a stopping criterion of three. The stopping criterion was tested after each successive interview. At the point when three successive interviews had been analysed without further thematic identification, data saturation was said to be achieved. As the final two interviews were carried out in succession, this criterion was exceeded by one. A sample size of 19 was therefore deemed sufficient.

Digitally recorded interviews were transcribed verbatim and de-identified, with participants assigned a participant identification number. All interviews were checked for accuracy (MLH) and the data were entered into the qualitative management program Nvivo v.9 (QSR International Pty Ltd, 2010) for analysis. Thematic analysis was applied to the data. Unlike other qualitative analysis methods (e.g. grounded theory, thematic discourse analysis, and interpretative phenomenological analysis) thematic analysis is not tied to a specific epistemology (e.g. it can be applied to data with a realist or social constructionist philosophy). As the aim of the research was to develop the meaning associated with the participants’ experiences, thematic analysis was deemed the most appropriate approach.

Data were coded by the first author (a PhD candidate with a background in psychology and post graduate training in qualitative research) following the procedure outlined by Braun and Clarke [40]. Briefly, finalised transcripts were systematically read and re-read prior to thematic coding to obtain an overall sense of the data. During this process, preliminary notes were made about the content of the text, particularly in terms of identified patterns and meanings within the data. Listening to the audio recording was accompanied on at least one read through of the transcript to verify that the meaning being attributed to the text was accurate.

Initial codes were then generated from the raw data. Transcripts were read in a line by line fashion, giving equal attention to each data item within the dataset, with sections of text relevant to the research entered into Nvivo as free standing ‘nodes’ (i.e. categories). Where possible, these initial nodes reflected the participants own words and all nodes were defined using descriptive labels. To maintain the context and meaning of the coded extracts, relevant surrounding data was retained [40]. At this stage of data identification and categorical organisation, substantive codes were generated, with text able to be coded in multiple nodes. This process ensured that repeated patterns within the dataset were adequately captured. Throughout the coding process, all transcripts were repeatedly reviewed and analysed. Similarities and differences were constantly compared to each other in an iterative fashion, with similar phenomena (or similar aspects of a phenomenon) grouped together. Comparisons were made within and across transcripts [41]. The refinement of higher order concepts (i.e. themes) involved ensuring that the generated codes formed coherent patterns within and across the datasets with disconfirming, as well as confirming evidence (i.e. deviant case analysis) sought [42,43]. This process ensured that the themes accurately reflected the lived experience of women with OA as a whole [40]. The content of the identified themes and example extracts were then reviewed, and discussed with the senior author (DL who has extensive experience in conducting qualitative research) until a consensus was reached. Themes were further refined by identifying the story or ‘essence’ contained within each theme in relation to both the research questions and overall data.

## Results

### Participant characteristics

A total of 44 women were invited to take part. In all, 25 women declined to participate. When provided, reasons for declining the invitation included undergoing treatments for other chronic illnesses (e.g. cancer) (n = 2), caring for an ill family member (n = 2), being too busy (n = 3) and lack of confidence in being able to contribute to the area of study (n = 4). The final participant sample included 19 women with an average age of 62.5 years at the time of the interviews (see Table 1). The majority of participants were Australian-born (n = 14), married or in cohabitating relationships (n = 14), living in rural areas (inner and outer regional areas) (n = 13), were either unemployed or retired (n = 9) and had achieved high school education or above (n = 15). Nine women reported being either unemployed or retired.

**Table 1:** Participant characteristics.

	Missing n (%)	Frequency n (%)
**Arthritis-related factors**		
**First reported arthritis diagnosis**		
Survey 3 (2001)		11 (57.9%)
Survey 4 (2004)		1 (5.3%)
Survey 5 (2007)		7 (36.8)
*Missing*	*0 (0*.*0%)*	
**Demographics**		
^*a*^ ^,^ ^*b*^ **Age**		62.5 (1.3)
*Missing*	*0 (0*.*0%)*	
**Marital status**		
Married/de facto		14 (73.7%)
Separated/divorced/widowed		4 (21.1%)
Never married		1 (5.3%)
*Missing*	*0 (0*.*0%)*	
**Area of residence**		
Urban		6 (31.6%)
Rural/remote		13 (68.4%)
*Missing*	*0 (0%)*	
**Educational attainment**		
Tertiary/post graduate		2 (10.5%)
Trade/diploma		6 (31.6%)
School/higher school certificate		7 (36.8%)
No formal education		2 (10.5%)
*Missing*	*2 (10*.*5%)*	
**Occupation**		
Highly skilled		4 (21.1%)
Skilled		2 (10.5%)
Less skilled		1 (5.3%)
No employment		9 (47.4%)
*Missing*	*3 (15*.*8%)*	
**Country of birth**		
Australia		14 (73.7%)
Other English speaking		2 (10.5%)
Europe		2 (10.5%)
Asia		1 (5.3%)
*Missing*	*0 (0*.*0%)*	

^a^ means and standard deviations are reported.

^b^ age at the time of the interview.

### The psychosocial impact of arthritis: “it affects everything you do, it affects everything you want to do”

OA was found to have a major impact, both physically and psychologically on the women interviewed. For some, pain was an ever-present entity *“mine’s a constant ache at times um it’s*, *it’s not sharp peaks of pain*, *it’s just that constant ache …”* [Participant 13]. For others, there appeared to be an ebb and flow, with the level of pain constantly changing and increasing in intensity. One participant noted that when her arthritis flared *“you just don’t know where to put your hand*, *you don’t know where to put the joint that’s hurting*, *it’s*, *you can’t describe it”* [Participant 19]. Factors related to pain persistence and symptom severity invoked negative emotions reflecting degrees of depression such as sadness, frustration and feeling a ‘bit down’. Feeling ‘sick of the constant pain’ or being depressed was also reported by some interviewees, *“Oh yes*, *I mean if [the pain’s] really bad*, *I do get a bit depressed”* [Participant 4].

Although the participants often described the psychological impact of arthritis increasingly impinging on their way of life in terms of ‘frustration’, the underlying theme ultimately revolved around sadness as a result of a ‘loss of independence’ and the ‘struggle to maintain a sense of self’ amidst the changing landscape of a *“cruel disease”* where *“you can see the changes happening”* [Participant 17]. As one participant noted *“a loss of depend*, *independence is something that is just*, *[it] eats away at you inside psychologically”* [Participant 19]. This emerged particularly as a result of conflicting self-concepts of who they were, and what they had achieved prior to OA, *“you become less of a person in your own eyes and ah*, *and that sort of um*, *I suppose gets me more frustrated …”* [Participant 19]. For most women, this inner conflict arose from perceived inadequacies associated with fulfilling their gender-specific caring roles, particularly as wives, mothers and grandmothers. As one participant suggested, *“Well I think you have a degree of depression when you’ve lost your … ability to do what you used to do*. *Very*, *very frustrating and I’ve always done all the handling*, *fixing*, *doing*, *whatevering …”* [Participant 11].

The struggle to maintain a ‘sense of self’ was often described by women as a *“constant fight”* [Participant 19] against feelings of *“being useless*, *… or being rendered … not able to do what you know you can do or should be able to do if [arthritis] wasn’t there*” [Participant 19]. It was particularly difficult for the women to watch the progressive and permanent physical deterioration reflective of an *“old”* person:

*You don’t like watching your*, *you know your body*, *I mean you can get big*, *you can get fat*, *you can get skinny and you can get wrinkly*, *but watching the bones deform as they go along* … *and you know that*, *that will continue*, *that won’t stop once it starts* [Participant 17].

A grief process often ensued following a series of ‘losses’ and recognition of increasing dependence on others:

… *you can’t do it*. *I think there is probably a period of*, *has been a period of grief at each stage where I have finally admitted that things are getting beyond me because I always had this concept of myself as I got older as being a slim active person who cares*. *I’ve always been a carer and to have to be cared for I find very difficult* … [Participant 14].

Some women also spoke about their anxiety concerning loss of physical capacity as they aged. One participant with severe OA noted that the stillness of the night was particularly difficult as this was a time when you were no longer able to *“just push it out of your consciousness”*. She often woke up with her stomach *“kind of seiz[ing] up”* and being *“petrified of what might be down the track”* [Participant 12]. Therefore, for women with OA, the emotional burden was high, with participants reporting a spectrum of emotions in relation to pain and increasing disability. The primary focus of this burden concerned the grief associated with the struggle to maintain a sense of identity in light of permanent physical deterioration.

### Psychological adjustment to arthritis over time: “you learn to live with it and … that’s basically it”

Despite fears regarding increasing arthritis-associated disability, the majority of women expressed acceptance of the disease, to some degree. It was suggested that despite it all, they had to *“learn to live with it”* [Participant 4]. This concept emerged as an important contributor to long-term psychological adjustment. Therefore, while the notion of *“you’ve got to accept it*. *It’s a part of life”* [Participant 9] was a common thread throughout the interviews, the acceptance of increasing limitations and disability appeared to be a dynamic ‘day by day’ process. This consisted of grieving physical losses and increasing dependence whilst managing daily chronic stressors such as pain, followed by a period of psychological readjustment. One participant described this as *“reluctant acceptance”* [Participant 14]. Over time, this balancing process was facilitated by a number of factors. These approaches primarily concerned personal beliefs about the disease, in conjunction with cognitive strategies that facilitated the acceptance of pain or the reappraisal of arthritis in the context of their lives as a whole.

### Personal beliefs and attitudes

Personal beliefs and attitudes were identified as significant to women coming to terms with their deteriorating body. Beliefs surrounding the permanence of the condition, viewing OA as an uncontrollable entity reflective of the ageing process and perceiving resistance to physical changes as futile were important. In spite of the reality of the situation, women chose not to focus on the inevitable disease progression and remained ‘stoic’ in their approach to life (i.e. displaying self-control in the face of arthritis-related stressors and remaining unemotional in order to overcome potentially destructive emotions).

**Stoicism: “just get on with it.”** Women with OA tended to employ a stoic attitude towards arthritis with phrases *“just get on with it”* and *“I just don’t let it get to me”* uttered by the majority of women. This coping style seemed to be born out of generational and gender role attitudes and transferred to other areas of their lives, *“that’s how you were brought up in my time*. *Suck it in and get on with it”* [Participant 3]. Stoicism often coincided with the participants behaving physically as they had done prior to having OA or ignoring key arthritis-related symptoms (often to their own detriment), suggesting that by definition having the disease meant that *“you have to learn to live with a certain amount of pain”* [Participant 12].

**Reasons for having arthritis: “they seem to think it’s old age, don’t they?”** Possessing certain beliefs regarding the cause of OA assisted participants with psychologically adjusting to the effects of arthritis. Women implicated a range of factors including accidents and falls, sporting injuries, dancing as a child, as well as repetitive strain from physically demanding occupations and gender roles as some of the reasons for their arthritis. A number of women had a family history of arthritis and as such viewed arthritis as a consequence of *“genetic garbage”* [Participant 11]. The majority of participants also described OA, as simply *“wearing out”* and a natural part of the ageing process. Women accepted that this process would be ongoing, with the aches and pains a signal of *“another bit break[ing] down”* [Participant 3]. Acknowledging arthritis as natural was buoyed by knowing others with the *“common”* disease. Despite the negative nature of OA, group membership appeared adaptive in terms of disease acceptance and psychological adjustment.

**Perceptions regarding symptom onset: “it’s a gradual thing.”** Women with OA perceived that the gradual onset of symptoms and limitations fostered a sense of resilience against the potential deleterious impact of the disease. Described as a *“slow creeping disease”* [Participant 14], this allowed women to incrementally manage this chronic stressor as the disease progressed. The gradual increase in pain intensity further assisted the participants cognitively in terms of developing pain-coping strategies and by extension aided psychological adjustment through allowing a tolerance to pain to be constructed over time.

*If this had leapt on me straight up*, *I probably wouldn’t have known what hit me*, *I would’ve gone to the doctor and said*, *you know I’m dying [laughs] because it’s a gradual thing and that the intensity has increased as I’ve gotten older*, *um I think I’ve built up a tolerance to the level of pain and as I said it’s only when it’s excruciating now that it really stops me from doing things*, *or otherwise I work through it* … [Participant 19].

### Cognitive factors

Long-term psychological adjustment to OA was however primarily facilitated by cognitive processes. These involved active and passive attempts to modify thoughts and feelings associated with arthritis-related stressors. Cognitive approaches included accepting arthritis-associated limitations, focusing on the positive and being grateful for what could still be achieved as well as having someone to measure their arthritis against (i.e. making downward comparisons).

**Accepting arthritis-related pain and limitation: “you can’t do what you used to do.”** Acknowledging new limits to physical abilities and constantly adapting tasks helped for a number of women adjust over time. Women acknowledged that OA involved having both good and bad days and understood that *“you can’t do everything”* [Participant 4]. One participant expressed that adjustment involved a gradual process of learning to *“listen to your body”* [Participant 6] and identifying physical limits. It was also important to *“lower your own expectations of what you can do”* otherwise *“you can set yourself up for disappointment all the time”* [Participant 19]. Maintaining high expectations of what can still be accomplished physically was suggested as a threat to psychological adjustment.

**Pain minimisation and comparative coping: “I’m not crippled with it or anything like that.”** The perceptions women held about arthritis assisted with psychological adjustment. Participants used pain minimisation techniques and comparative coping strategies in order to minimise the severity of their illnesses. Pain, and its impact, was often described using minimising language such as *‘niggle’*, *‘ache’*, *‘nuisance’*, *‘annoyance’*, and a *‘little bit of pain’* with women suggesting that *“you’ve just gotta put up with your lot in life …”* [Participant 18]. This was despite sometimes significant arthritis-associated disability. Women also compared themselves against other people with arthritis, including friends, relatives and individuals within the community. *“Being crippled”* was often used as a point of reference *“it’s not as if I’m crippled with it you know*. *It slows me down but you know [it’s] nothing compared to what heaps have got”* [Participant 5]. Having someone to measure their arthritis against appeared to be critical for minimising the impact of the disease. Women also compared themselves to individuals with conditions perceived to be more debilitating such as heart disease or cancer. Cancer in particular was viewed as having a *“death sentence”*, and given the choice between having arthritis or cancer, “*they[‘d] rather have arthritis”* [Participant 18]. Comparisons were not restricted to those around chronic disease One participant compared the difficulties associated with arthritis to global events, such as earthquakes, suggesting that there were many people less fortunate and that it was important not to *“grizzle about a few aches and pains”* and to be *“thankful for what you’ve got”* [Participant 5]. Thus, the women’s perceptions of arthritis were also shaped by their outlook on life.

**Cognitive reappraisal: “I don’t take things for granted anymore.”** Psychological adjustment to OA for some women was facilitated by cognitively reappraising their situation and finding personal growth in their experiences. Having arthritis assisted the participants in developing a greater awareness and appreciation for the struggle others face, as well as gaining an awareness of their bodies and having greater attunement to their physical limitations. Enduring chronic pain was said to be beneficial to personal growth, with it likened to a muscle that requires exercise, *“*… *if you have nothing to endure you never get to build up that quality in yourself … the pain of arthritis teaches you at least that you can endure …”* [Participant 19]. This ability appeared to be enhanced by the maturity gained with older age.

Moreover, psychological adjustment was facilitated by redefining perceptions about specific life stages and self-concepts (whilst maintaining control over arthritis symptoms). Despite the significant emotional burden associated with the physical aspects of the disease, the participants found ways in which to mitigate the daily concerns associated with arthritis, and developed strategies over time in an attempt to integrate the disease into their identity. Maintaining a meaningful existence and a sense of independence for as long as possible by finding *“something else to become passionate about”* [Participant 15], was also paramount. Psychological adjustment ultimately concerned relinquishing perceptions about past abilities and ‘creating a new normal’ *“you can go back to your normal life … you just have to learn to live like that …”* [Participant 12].

## Discussion

Arthritis is an insidious and pervasive chronic condition that significantly impacts on mental health and physical functioning [1]. This study aimed to qualify the psychosocial burden and examine factors contributing to long-term psychological adjustment for women with OA. The findings indicate that for older women, the emotional burden of OA is considerable, and the process of psychological adjustment complex. This study adds important information to the wider body of knowledge surrounding arthritis-related psychosocial challenges for women as they age.

Considerable evidence exists to suggest that arthritis and mental health are intimately intertwined [17,44,45]. For women in this study, the emotional burden of arthritis was found to primarily involve the experience of pain, coupled with increasing loss of function and resultant disability. Pain and increasing disability have previously been reported as the prime psychosocial stressors confronting middle-aged and older individuals with arthritis [46,47]. It has been suggested that arthritis-related pain and limitation presents a significant challenge to the individual’s sense of self, impacting upon the way in which they perceive themselves, interact with others, as well as their ability to carry out valued roles [48,49]. This notion is supported by the findings of the current study. Particularly for older women, significant distress was associated with the decreased ability to run the household, care for ailing spouses and parents, and participate in the care of grandchildren. Restrictions in these life spheres facilitated feelings of loss, inadequacy and anxiety surrounding increasing dependence. Lutze and Archenholtz [27] in their RA-focused study found that fears regarding the long-term impact of the disease presented early. For women in this study who reported OA, fears regarding the loss of independence appeared later, coinciding with disease progression.

Adjustment to OA over the long-term was revealed as a dynamic ‘day to day’ process involving a constant struggle between grieving physical losses and increasing dependence amidst symptom management. This process of ‘reluctant acceptance’ has also been reported in relation to long-term psychological adjustment to RA [30,50] and ankylosing spondylitis [51]. Iaquinta and Larrabee [50] in a small phenomenological analysis of women aged 47–67 described the struggle with this process as ‘grieving whilst growing’. Grieving promoted personal growth by allowing individuals to rise above their personal circumstances. Losses were reconciled with the grief attached to those losses not impinging on future desires or achievements. Coping with arthritis did not represent an endpoint of adjustment, but revolved around ongoing cognitions in which the participant had to reframe their self-concept at each stage of the disease process. Plach et al. [52], in a sample of adults aged 39–86 years indicated that the process of relinquishing was an important concept in providing women with a sense of control that offset such losses. The findings of this OA-dominant study, coupled with previous RA-focused research, suggest that the process of adjustment does not appear to be contingent on the type of arthritis experienced by women.

While cognitive mechanisms were pivotal to the reconciliation of perceptions associated with dependence and independence in this study, psychological adjustment to OA over time was often facilitated by ingrained belief systems and attitudes. The majority of participants minimised their symptoms, viewing OA as a natural and permanent artefact of the ageing process. OA was simply labelled a consequence of “wear and tear”. This notion is supported by other qualitative studies primarily involving older individuals with severe OA symptoms [48,53,54], and contrast with the view of Ailinger and Schweitzer [46] who argued that the cause of the disease was not important to arthritis patients. Possessing such views has been found to provide a barrier to seeking treatment [55]. Gignac and colleagues [48] suggested that this perception was perpetuated by health professionals, with physicians dismissing arthritis-related symptoms. With perceptions surrounding OA symptoms as ‘normal’ pervasive, this may have contributed to feelings of a lack of entitlement, and as such resulted in the minimisation of pain. Maintaining a stoic attitude and making downward comparisons about their condition or functionality with other individuals may have been an adaptive mechanism in this instance.

The complexities surrounding adjustment to OA have been a relatively under-researched phenomenon using qualitative methods. While RA is commonly viewed as a more insidious disease due to its inflammatory component, these findings suggest that the adjustment process for women with OA is similar to that of RA. These findings add to the wider body of knowledge surrounding psychological adjustment to arthritis and have the potential to inform clinical interventions and alter disease trajectories for women. The results suggest that focusing on clinical interventions aimed at assisting women with OA come to terms with increasing disease-related disability are warranted. Sharpe and colleagues [56] found that a lack of coping skills significantly predicted depression in older adults with arthritis. Such efforts may assist in fostering resilience in the face of inevitable physical deterioration. Sturgeon and Zautra [57] suggested that resilient individuals adopt more adaptive strategies to pain, possess a greater belief in their abilities to effectively control pain, possess greater emotional knowledge, and direct more attention to evaluating their own current emotional state. This type of therapy would also assist with reappraising and finding meaning in the arthritis experience, and thus facilitate the acceptance of, or encourage appropriate control of pain. Public health education strategies aimed at the general population are also required to dispel misconceptions surrounding chronic joint symptoms and the ageing process. The synergy between the findings of this study and that of Gignac et al. [48] suggest that these perceptions are already present by middle age. Highlighting the destructive nature of OA, as well as providing education regarding pain being unnatural and not a consequence of “wear and tear”, may assist with increased early detection and treatment in middle-aged and older adults. Such practices have the potential to modify disease trajectories.

Trustworthiness of the research was evaluated according to the criteria suggested by Kitto and colleagues [58] and was conducted in accordance with the consolidated criteria for reporting qualitative research [59]. Factors such as purposefully sampling participants, creating transparency at each stage of the process (such as a comprehensive description of the decisions and procedures involved in the collection, recording and analysis of the data including the maintenance of field notes and researcher triangulation) as well as creating an ‘audit trail’ that may be subject to external scrutiny contributed to the study’s rigour. In addition, a systematic process was used to achieve data saturation [39]. These approaches allowed for the corroboration of previous arthritis research and generated novel findings that will provide avenues for further investigation.

While this qualitative analysis was able to examine psychological adjustment to OA in-depth, it must be considered in light of a few limitations. Due to the delay between symptom onset and diagnosis of arthritis, the length of time since arthritis diagnosis could not be determined. However, participants reported having been diagnosed with OA more than four years prior to the interview and we were able to examine a breadth of experiences. Additionally, these findings must be evaluated within the context of participants’ lives as a whole. The experience of psychosocial stress in other life spheres may have potentially contaminated their experiences of arthritis. Further, the findings may not be representative of the experiences of men. With women in this age group twice as likely as men to develop arthritis, and the psychosocial sequelae for women significantly higher, this cohort is appropriate [60,61].

## Conclusion

The findings of this OA-focused qualitative study add to the current understanding surrounding the psychosocial impact of OA and the complex processes involved in psychological adjustment over time. Importantly, psychological adjustment over time was attributed primarily to cognitive and attitudinal factors. This process was a dynamic ‘day to day’ process involving a constant struggle between grieving physical losses and increasing dependence amidst symptom management. Developing accurate quantitative measures to assess the complexity involved in adjustment are required to confirm these findings at a population level. By understanding the destructive and preventable nature of arthritis, this may facilitate early detection and increased uptake of appropriate treatment options for OA that not only provide symptom relief but have the ability to modify disease trajectories. In doing so, this will not only reduce the economic burden associated with the disease, but will also facilitate women in ageing well.

## Supporting Information

S1 Table Semi-structured interview schedule. The interview schedule formed the basis for examining two separate research questions. The sections *“Psychosocial impact of being diagnosed with arthritis”* and *“looking to the future”* are most relevant to understanding the psychosocial impact of osteoarthritis for older women.
(DOCX)
