# Supplementary material for: “Just Get on with It”: Qualitative Insights of Coming to Terms with a Deteriorating Body for Older Women with Osteoarthritis
Source: PLoS One. 2015 Mar 17;10(3):e0120507. doi: 10.1371/journal.pone.0120507 (PMC4364122; doi:10.1371/journal.pone.0120507)
Supplement: S1 Table — The interview schedule formed the basis for examining two separate research questions. The sections “Psychosocial impact of being diagnosed with arthritis” and “looking to the future” are most relevant to understanding the psychosocial impact of osteoarthritis for older women. (DOCX) [file pone.0120507.s001.docx]

**S1 Table. Semi-structured interview schedule.** The interview schedule formed the basis for examining two separate research questions. The sections *“Psychosocial impact of being diagnosed with arthritis”* and *“looking to the future”* are most relevant to understanding the psychosocial impact of osteoarthritis for older women.

| **Question** | **Prompt** |
| --- | --- |
| ***Psychosocial impact of being diagnosed with arthritis*** | |
| - Today I’d like to ask you about your life experiences and your experiences with living with arthritis. Firstly, could you describe your experience of being diagnosed with arthritis? | - You could start by describing how you came to be diagnosed? - What things were happening for you that led you to seek medical advice? |
| - What was going on for you in your life at the time of being diagnosed? | - Was there anything major happening in your life at the time or daily hassles such as issues with money? - How were things with your friends or family? - **What is going on for you in your life when your arthritis/symptoms are worse (e.g., when you have pain, fatigue or stiffness)?** - **What makes it harder for you to cope with having arthritis or dealing with your symptoms? What makes it easier?** |
| - How has your life been affected by arthritis? | - How has having arthritis affected you physically? Emotionally? With your relationships with friends or family? |
| - How has your ability to cope with arthritis changed from when you were diagnosed until now? | - How do you feel about how things have changed for you? |
| ***Psychosocial impact prior to arthritis diagnosis*** | |
| - Now, I’d like to ask you about your life prior to being diagnosed with arthritis. Could you tell me what your life was like before you began experiencing symptoms? | - Thinking about your life growing up and in your early adult years, how would you generally describe the experiences in your life? - Have you experienced one or a series of stressful events or have they been on the whole positive? - What kind of things did you do to cope? - What kind of impact do you think this/these event(s) had on your health (physically and emotionally)? - What are your thoughts and feelings about this time in your life now? |
| ***Understanding of the relationship between stress and health*** | |
| - Switching focus now, I’d like to ask you about experiencing stress in relation to your health. How do you think stress affects your health (physically and emotionally)? | - How often would you say that you get stressed? - What is happening in your life when you feel stressed? - How do you know when you are feeling stressed? - What do you do to cope when you are feeling stressed? - **How do you respond to feeling stressed now compared to before having arthritis? Compared to when you were younger?** |
| ***Looking to the future*** | |
| - To finish up, I’d like you to now reflect on how your life has changed since being diagnosed with arthritis. How is it different? |  |
| - Is there anything you would like to add that we haven’t already discussed? |  |
